# Neuroligin-2 shapes individual slow waves during slow-wave sleep and the response to sleep deprivation in mice

**DOI:** 10.1186/s13229-024-00594-5

**Published:** 2024-04-03

**Authors:** Tanya Leduc, Hiba El Alami, Khadija Bougadir, Erika Bélanger-Nelson, Valérie Mongrain

**Affiliations:** 1 Department of Neuroscience, Université de Montréal, Montreal, QC Canada; 2 Centre d’études avancées en médecine du sommeil (CÉAMS), Recherche - Centre intégré universitaire de santé et services sociaux du Nord-de-l’Île-de-Montréal, Montreal, QC Canada; 3 Centre de recherche du Centre hospitalier de l’Université de Montréal, 900, St-Denis street, Tour Viger Montréal, Montreal, QC H2X 0A9 Canada; 4 Present Address: Pfizer Canada ULC, Montreal, QC Canada

**Keywords:** GABAergic neurotransmission, Synaptic adhesion molecules, Sleep-wake regulation, Sleep deprivation, Slow waves, Gene expression, Cerebral cortex, Mice

## Abstract

**Background:**

Sleep disturbances are a common comorbidity to most neurodevelopmental disorders and tend to worsen disease symptomatology. It is thus crucial to understand mechanisms underlying sleep disturbances to improve patients’ quality of life. Neuroligin-2 (NLGN2) is a synaptic adhesion protein regulating GABAergic transmission. It has been linked to autism spectrum disorders and schizophrenia in humans, and deregulations of its expression were shown to cause epileptic-like hypersynchronized cerebral activity in rodents. Importantly, the absence of *Nlgn2* (knockout: KO) was previously shown to alter sleep-wake duration and quality in mice, notably increasing slow-wave sleep (SWS) delta activity (1–4 Hz) and altering its 24-h dynamics. This type of brain oscillation is involved in memory consolidation, and is also a marker of homeostatic sleep pressure. Sleep deprivation (SD) is notably known to impair cognition and the physiological response to sleep loss involves GABAergic transmission.

**Methods:**

Using electrocorticographic (ECoG) recordings, we here first aimed to verify how individual slow wave (SW; 0.5-4 Hz) density and properties (e.g., amplitude, slope, frequency) contribute to the higher SWS delta activity and altered 24-h dynamics observed in *Nlgn2* KO mice. We further investigated the response of these animals to SD. Finally, we tested whether sleep loss affects the gene expression of *Nlgn2* and related GABAergic transcripts in the cerebral cortex of wild-type mice using RNA sequencing.

**Results:**

Our results show that *Nlgn2* KO mice have both greater SW amplitude and density, and that SW density is the main property contributing to the altered 24-h dynamics. We also found the absence of *Nlgn2* to accelerate paradoxical sleep recovery following SD, together with profound alterations in ECoG activity across vigilance states. Sleep loss, however, did not modify the 24-h distribution of the hypersynchronized ECoG events observed in these mice. Finally, RNA sequencing confirmed an overall decrease in cortical expression of *Nlgn2* and related GABAergic transcripts following SD in wild-type mice.

**Conclusions:**

This work brings further insight into potential mechanisms of sleep duration and quality deregulation in neurodevelopmental disorders, notably involving NLGN2 and GABAergic neurotransmission.

## Introduction

Sleep disturbances are predominant in patients suffering from neurodevelopmental disorders and often associated with worse disease outcomes. Decreased sleep duration, increased sleep fragmentation, and alterations in electroencephalographic activity have notably been reported in autism spectrum disorders (ASD) and schizophrenia [1–5]. Importantly, less total sleep time was correlated with worse social and communication skills in patients with ASD [2]. Likewise, poorer sleep hygiene and sleep spindle deficits were associated with worse positive/negative symptoms and cognitive functions in patients with schizophrenia [5, 6]. Epileptic syndromes also present with numerous sleep disturbances, which can include less time spent in paradoxical sleep (PS) and/or longer PS latency [7–10], and increased subjective sleepiness [11]. Sleep complaints were even found to associate with a worse quality of life in epileptic patients [12]. Accordingly, understanding the molecular mechanisms underlying sleep disturbances in neurodevelopmental disorders could help alleviate disease symptomatology.

Neuroligins (NLGNs) are adhesion proteins usually expressed at postsynaptic sites and involved in both neurodevelopment and synaptic function [13, 14]. Neuroligin-2 (NLGN2), which mainly regulates gamma aminobutyric acid (GABA)ergic neurotransmission, but also cholinergic and dopaminergic transmission [15–18], was repeatedly associated with neurodevelopmental disorders. In humans, mutations in *NLGN2* have been linked to ASD and schizophrenia [19, 20], while rodents with NLGN2 deregulations have been found to have behavioral impairments, such as anxiety-like behaviors and impaired social interactions [21–23]. Moreover, both knocking out (KO) and overexpressing *Nlgn2* in mice was shown to cause abnormal (potentially epileptic-like) hypersynchronized cerebral activity [21, 24, 25]. Interestingly, our group previously reported important sleep disturbances in *Nlgn2* KO mice, such as decreased time spent asleep and a wide range of changes in electrocorticographic (ECoG) activity during wakefulness and sleep states [24]. Thus, NLGN2 represents a good candidate to unravel the etiology of sleep disorders in neurodevelopmental pathologies.

During slow-wave sleep (SWS), cortical neurons alternate between a hyperpolarized/silent (down) state and a depolarized/firing (up) state, creating slow waves (SW) of low frequency (< 4 Hz) and high amplitude [26, 27]. SW and slow-wave activity, which are implicated in cognitive functions including memory consolidation and extinction [28, 29], were reported to be altered in autistic and epileptic patients [30–32]. Our previous findings show a large increase in SWS delta (1–4 Hz) activity in *Nlgn2* KO mice [24]. However, it remains to be defined whether this effect originates from an increased capacity to generate SW during SWS (increased density) or in more synchrony between individual neurons contributing to SW generation (higher amplitude and steeper slope). This is of relevance to help define the role of NLGN2 in SW generation during SWS as well as in sleep-related cognitive alterations observed in neurodevelopmental disorders.

Sleep deprivation (SD) was shown to impact GABAergic neurotransmission in key sleep regulatory areas of the rodent brain [33–36]. Given the involvement of NLGN2 at GABAergic synapses [15, 16], and that increased immunostaining of NLGN2 was reported after SD on wake-promoting orexin neurons [33], the KO of *Nlgn2* in mice might affect the response to sleep loss (e.g., sleep duration and distribution, ECoG activity). Our observation that *Nlgn2* KO mice have an altered 24-h dynamics of SWS delta activity under baseline (BL) conditions [24] also supports this hypothesis. Indeed, the dynamics of SWS delta activity is well-recognized to reflect sleep homeostasis, being increased by prolonged wakefulness and dissipating during sleep [37, 38]. The altered 24-h dynamics of SWS delta activity in *Nlgn2* KO mice could thus indicate potential dysfunctions in homeostatic sleep regulation, which can be further investigated by assessing the response to SD. Interestingly, an impairment of SW slope dissipation across the night was observed in epileptic patients [30], and SD was reported to increase the occurrence of epileptic discharges and to trigger seizures [39]. Considering the above-mentioned epileptic-like ECoG activity observed under abnormal expression of *Nlgn2* in mice [21, 24, 25], this further underscores the relevance of investigating the involvement of NLGN2 in the response to sleep loss.

We here tested the first hypothesis that different SW properties would be altered during SWS in *Nlgn2* KO mice. Given the elevated SWS delta activity in these mutant animals [24], we specifically expected to find SW of higher amplitude and steeper slope, in addition to a higher density of SW per minute of SWS. Our second hypothesis was that KO mice would show modifications in their response to SD both at the level of vigilance state duration and ECoG activity. We further hypothesized that hypersynchronized event occurrence would be increased in the course of SD in KO mice, peaking at the end of the SD. To test these hypotheses, the ECoG of *Nlgn2* KO mice and littermates was recorded during undisturbed/BL conditions and following a 6-h SD, and the ECoG signal was submitted to state identification, spectral analysis and SW detection. Our findings support the first two hypotheses, revealing multiple alterations in SW properties during SWS and modifications in sleep duration and quality after enforced wakefulness in the absence of NLGN2. However, hypersynchronized events did not show the anticipated changes during SD. We additionally verified the effect of a 6-h SD on *Nlgn2* expression in the cerebral cortex of WT mice and found it to be decreased, along with the expression of many other GABAergic transcripts. Altogether, these results bring further insight into basic sleep-wake regulatory mechanisms involving adhesion molecules, which could help clarifying the etiology of sleep disturbances in neurodevelopmental disorders.

## Methods

### Animals

Male adult mice of mixed genetic background (B6;129-*Nlgn2*^*tm1Bros*^/J) had their ECoG signal recorded under both BL and sleep deprived conditions (48 h continuous recording). The BL part of the recording (first 24 h) was previously analyzed for wake/sleep architecture and ECoG activity [24], but not for SW properties and density. We here analyzed SW of the BL ECoG signal of the same animals, and also newly investigated their response to SD. Breeding couples were initially purchased from Jackson Laboratories and bred to obtain *Nlgn2* wild-type (WT), heterozygous (HET), and KO mice. The KO of *Nlgn2* was previously generated by homologous recombination specifically targeting the first coding exon of the mouse *Nlgn2* [40]. The current study includes 14 WT mice (age and weight at time of surgery: 69.2 $\pm$ 2.0 days, 27.5 $\pm$ 0.6 g), 13 HET mice (71.6 $\pm$ 2.1 days, 27.1 $\pm$ 0.8 g), and 12 KO mice (70.8 $\pm$ 2.0 days, 24.3 $\pm$ 0.8 g). This slightly differs from our earlier analyses (14 WT, 14 HET and 12 KO) [24], given the exclusion of one HET mice due to an abnormal signal during the SD recording. As previously, the three groups did not significantly differ for age (F_2,36_ = 0.37, *p* = 0.7), but *Nlgn2* KO mice weighted significantly less (F_2,36_ = 6.0, *p* = 0.006) than both WT (*p* = 0.003) and HET (*p* = 0.009) mice. Two weeks before surgery, mice were transferred to individual cages. Additional C57BL/6J male WT mice (*n* = 18; aged 81.7 $\pm$ 0.7 days) were used for RNA sequencing. All mice were kept under a 12-h light/12-h dark cycle, at a room temperature maintained between 23 and 25 °C, and with food and water freely accessible throughout the experiment. All procedures have been conducted according to guidelines of the Canadian Council on Animal Care, and have been approved by the *Comité d’éthique de l’expérimentation animale* of the *Centre intégré universitaire de santé et services sociaux du Nord-de-l’Île-de-Montréal*.

### Surgery and electrophysiological recording

ECoG and electromyographic (EMG) electrode implantation surgery was performed as previously described [41–43], when mice reached 9–10 weeks of age. These surgeries were performed under deep ketamine/xylazine (120/10 mg/kg, intraperitoneal injection) and isoflurane (0.5-1%) anesthesia. Briefly, mice were installed in a stereotaxic frame, and three gold-plated screws of 1.1 mm diameter were screwed through the skull over the right hemisphere to serve as ECoG electrodes (anterior electrode: 1.5 mm anterior to bregma and 1.7 mm lateral to midline; posterior electrode: 1.0 mm anterior to lambda and 1.7 mm lateral to midline; reference electrode: 0.7 mm posterior to bregma, 2.6 mm lateral to midline). Three anchor screws were implanted over the left hemisphere to support the montage, and two gold wires were inserted into neck muscles to serve as EMG electrodes. All electrodes were soldered to a connector and the montage was secured on the skull with dental cement. Approximately four days after surgery, mice were cabled and connected to a swivel contact. They were habituated to cabling conditions for another week before recording.

Ten days after electrode implantation, ECoG and EMG signals were continuously recorded for 48 h starting at light onset (Zeitgeber time 0: ZT0) including 24 h of BL, 6 h of SD, and 18 h of recovery. SD was performed by gently disrupting the litter next to the mice when they adopted a sleeping behavior (gentle handling). Mice were transferred to a cage with clean litter at the beginning of the fourth hour of the SD, and paper tissues were given during the third and last hours of the SD only when necessary to keep the mice awake. Electrophysiological signals were amplified using Lamont amplifiers, and sampled at 256 Hz with the Stellate Harmonie software (Natus, San Carlos, CA). Recordings were segmented in 4-s epochs offline to conduct visual identification of vigilance states (i.e., wake, SWS, and PS) based on ECoG and EMG features as previously described [44]. The bipolar ECoG signal between the anterior and posterior electrodes was used for vigilance state identification, SW detection, and spectral analysis.

### SW detection and analyses

SW detection was conducted on the bipolar ECoG signal of artefact-free SWS epochs for the whole 48 h of recording (BL, SD, and recovery). An initial manual detection of SW (minimum peak-to-peak amplitude of 120 $\mu$V; minimum total duration of 0.25 s) was performed on the raw signal of six 1-h recording sections from both WT (*n* = 4) and KO (*n* = 2) mice to optimize automatic detection parameters. The signal was then band-pass-filtered between 0.5 and 4.0 Hz with a linear phase FIR filter of -3 dB for automatic detection. Automatic detection was first performed on the filtered signal of these 6 recording sections using previously published criteria [45, 46]: peak-to-peak amplitude > 120 $\mu$V, negative peak amplitude > 40 $\mu$V, negative phase duration between 0.1 and 1 s, and positive phase duration < 1 s. Final automatic detection was optimized by changing the minimum peak-to-peak amplitude criteria (> 100 $\mu$V). This optimization was guided by concordance analyses between manual and automatic detections, which considered precision (fraction of automatically detected SW that were also manually identified), recall (fraction of manually identified SW found by the detector), and a F1 score (i.e., 2 x [precision x recall] / [precision + recall]; see [47]). The optimized detection resulted in a modest increase in precision (from 77.6 to 78.3%), and in notable increases in both recall (from 73.7 to 82.8%) and F1 score (from 0.74 to 0.8). Interestingly, the optimization mainly increased the recall and F1 score for WT mice, while having little effect on SW detection in KO mice.

SW density (number of SW per minute of SWS) and properties were compared between genotypes for the filtered signal of the 24 h of BL and 24 h of SD and recovery. SW properties included peak-to-peak amplitude, slope (velocity of the change from the negative peak to the positive peak), negative and positive phase durations, and frequency (1/ total SW duration). For BL analyses, SW density and properties were averaged for 12 equal intervals during the light (resting) period, and 6 equal intervals during the dark (active) period, as previously performed [45, 46]. For SD and recovery analyses, they were averaged for 8 equal intervals during the 6 h of the light period after SD, and for 6 equal intervals during the subsequent dark period (see also [45, 46]). For each mouse, an equal number of SWS 4-s epochs contributed to each interval within the light and dark periods of each recording days. SW density and properties were also expressed as a percentage of BL mean values to emphasize SD-induced changes from BL.

### Sleep architecture and spectral analysis

The percent time spent in each vigilance state during SD and recovery was averaged for the total 24 h, the 12-h light period and the 12-h dark period. The time spent in each vigilance state was then calculated per h across the 24 h. The number and the mean duration of individual bouts of vigilance states were also computed for the light and dark periods. The time spent in SWS or PS during SD, and the latency to SWS or PS after SD, were computed. The accumulated SWS and PS differences from BL were calculated across 24 h using the previously published time courses [24]. SWS and PS recovery slopes were extracted from these time courses between the 6th and the 12th intervals. These intervals were chosen to target the initial period during which animals recover their sleep after SD, before the dark (active) period.

A fast Fourier transform was applied on the bipolar ECoG signal of the SD and recovery 24-h day to calculate ECoG spectral activity of each vigilance state between 0.5 and 50 Hz with a 0.5 Hz resolution. Absolute power was then normalized for each mouse by dividing the activity of each 0.5 Hz-bin by the BL mean total activity (all Hz-bin of all vigilance states). To emphasize SD-BL differences, relative power spectra were then expressed as a percentage of BL data (i.e., the normalized activity of each Hz-bin was expressed as a percentage of the corresponding BL Hz-bin normalized activity). Finally, the time course of spectral activity in specific frequency bands was investigated. More precisely, wake theta (6–9 Hz), alpha (9–12 Hz) and low gamma (30–50 Hz) activity were computed over 8 equal intervals during SD, 3 equal intervals during the remaining 6 h of the light period, and 12 equal intervals during the subsequent dark period (similar to [42, 48]). SWS delta (1–4 Hz) activity was computed over 8 equal intervals during the 6 h of the light period following SD, and over 6 equal intervals during the subsequent dark period [42, 48, 49] similar to SW density and properties. To assess the dynamics within animals without the confounding effect of large between-mouse differences, spectral activity in each interval was expressed as a percent of the 24-h BL mean for each frequency band separately.

### Hypersynchronized events

Hypersynchronized events were manually detected on 24-h ECoG traces of SD and recovery according to the same two criteria as our previous work: an amplitude of at least twice that of background activity, and a minimum duration of one second [24]. Background ECoG amplitude was visually determined for each vigilance state, and adapted for each mouse. Events separated by no more than 0.5 s were considered as one. The number of events occurring during wake and PS were separately calculated for the light and dark periods, as well as for each h of the 24-h SD/recovery recordings. To control for the time spent in each vigilance state, a density was calculated by dividing the number of wake and PS events of the light and dark periods by the time spent in these respective states. The density of events was also calculated individually for each h of the 24-h SD/recovery recording. The wake and PS event densities were also calculated for each h of the BL recordings with previously identified hypersynchronized events [24]. Importantly, as BL events were identified several years prior to the SD/recovery events and by different scorers, no direct comparisons of number/density of events, nor of event duration, was made between these conditions. The presented analyses focus on each 24-h recording conditions (i.e., BL and SD/recovery) separately, and assess 24-h dynamics in an attempt to unveil the effect of homeostatic sleep pressure on hypersynchronized event occurrence, in particular in the course of SD.

### Statistical analyses

For all time courses, two-way repeated-measure analyses of variance (ANOVA) were used with factors Genotype and Hour or Interval. Genotype differences in time spent in each vigilance state for the SD/recovery day (total 24 h, 12-h light period, and 12-h dark period), time spent in SWS/PS during the SD, latency to enter SWS/PS after SD, and SWS/PS recovery slopes were investigated using one-way ANOVA. Comparisons between genotypes for vigilance state individual bout duration and number for the 12-h light and dark periods were conducted with two-way repeated-measure ANOVA. Total 24-h spectral activity for the SD/recovery day was compared between genotypes for each Hz-bin using one-way ANOVA. Comparisons between genotypes for number and density of wake and PS hypersynchronized ECoG events for the light and dark periods of the SD/recovery and BL days were done using two-way repeated-measure ANOVA. Significant differences were decomposed using planned comparisons or post hoc Tukey’s tests. When performing repeated-measure ANOVA, statistical significance was adjusted with a Huynh-Feldt correction if needed. The threshold for statistical significance was set to 0.05, and data are reported as mean ± standard error of the mean.

### Gene expression quantification

The effect of SD on genome-wide gene expression in the mouse cerebral cortex was quantified using RNA sequencing (RNAseq) with a particular focus on *Nlgn2* and relevant genes (i.e., GABAergic, cholinergic, and dopaminergic synapse related genes). C57BL/6J male WT mice were either sleep deprived (*n* = 9) for 6 h starting at ZT0 by gentle handling (as described above) or left undisturbed (*n* = 9), and their cerebral cortex was sampled simultaneously at ZT6. RNA was extracted using a Qiagen RNeasy mini kit, and an equal amount of RNA from 3 separate mice was pooled into a single replicate (i.e., 3 replicates of 3 mice for both SD and control conditions; total of 400 ng per replicate). Samples were sent to the *Institut de recherches cliniques de Montréal* (Montreal, QC, Canada) for 50 bp paired-end RNAseq (Illumina Hi-Seq 2000). Base call (Illumina CASAVA 1.8 pipeline), read quality verification (FastQC v0.10.1) [50], low quality base removal (Trimmomatic v0.32) [51], and genomic alignment (Tophat v2.0.10) [52] were done by the *Institut de recherches cliniques de Montréal*. Quantification (HTSeq-count v0.5.3) [53], normalization (DESeq2 v1.4.5) [54], and statistical analyses (false discovery rate, FDR) [55] were also conducted by the bioinformatic platform of the *Institut de recherches cliniques de Montréal*.

Normalized read counts of differentially expressed genes (DEGs, FDR < 0.05) were plotted in a heatmap using RStudio and clustered with the Ward.D2 method (which searches for pairs that have minimum sum of squared errors upon merging). For clarity, transcripts with a non-significant difference between BL and SD were omitted. Normalized read counts were expressed in base 2 logarithm and scaled to each row’s mean value. The list of DEGs found in the two main clusters were separately fed to the Database for Annotation, Visualization and Integrated Discovery (DAVID) online tools (https://david.ncifcrf.gov/tools.jsp). The DIRECT biological processes, cellular compartments, and molecular function gene ontology (GO) libraries were used to obtain GO term charts. FDR < 0.05 were considered significant for GO term enrichment. Most relevant GO terms were plotted with their gene percentage, fold enrichment, and FDR. The gene percentage is the percentage of genes from the input sample that is involved in a given GO term. The fold enrichment is a ratio between this gene percentage and the expected percentage in the full exome.

## Results

### Altered SW under BL conditions in *Nlgn2* KO mice

**Fig. 1:**
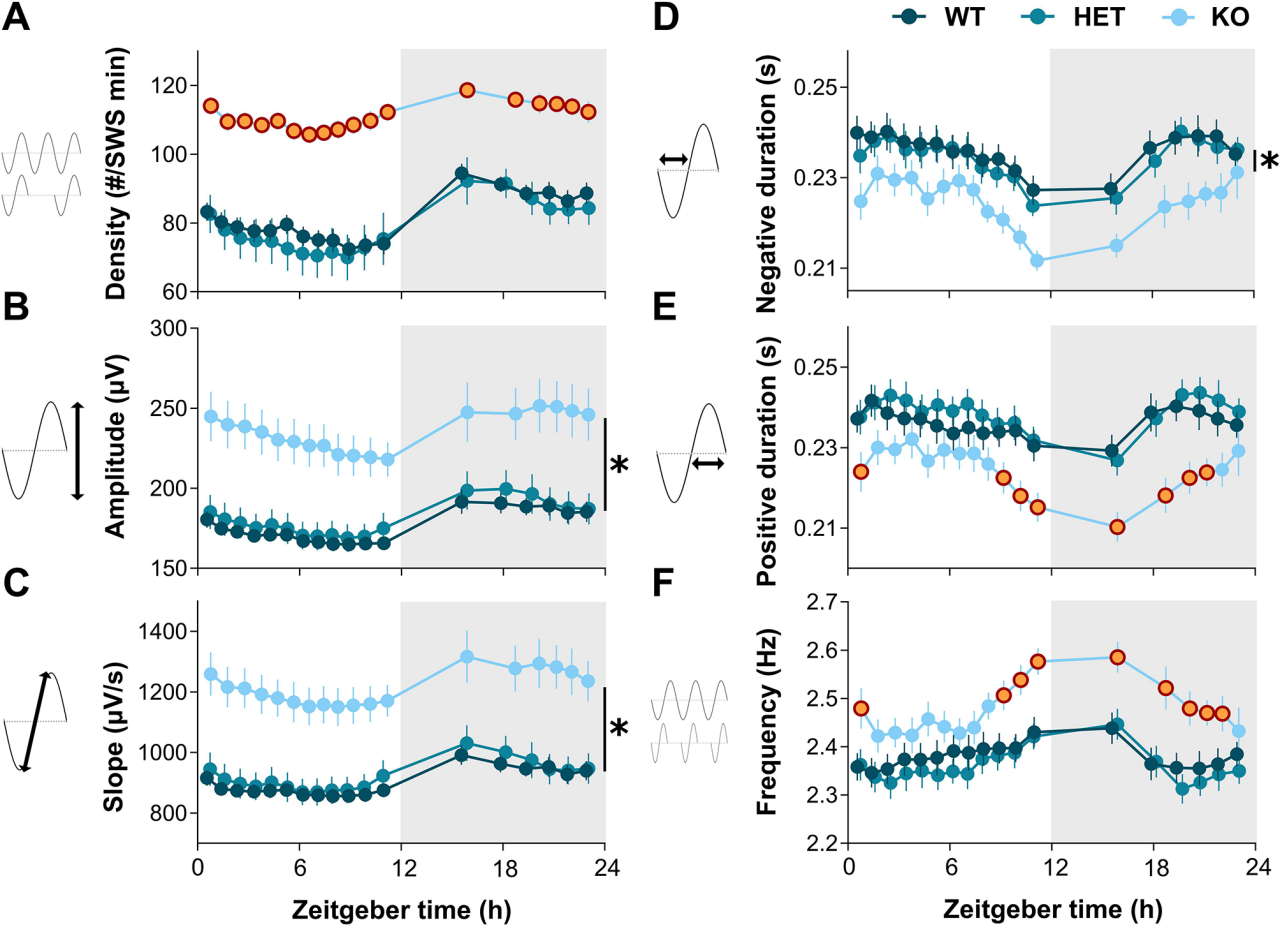
SW density and properties during SWS under BL conditions in *Nlgn2* KO mice and littermates. Twenty-four-hour dynamics of SW **(A)** density, **(B)** amplitude, **(C)** slope, **(D)** negative phase duration, **(E)** positive phase duration, and **(F)** frequency. Significant interactions between Genotype and Intervals were decomposed by planned comparisons and represented with different color datapoints. Significant main Genotype effects were decomposed by planned comparisons and illustrated with vertical lines accompanied by symbols. *Nlgn2* KO mice datapoints identified in orange with a red contour, as well as stars (*), indicate significant differences in comparison to HET and WT mice (*p* < 0.05). Gray backgrounds represent the dark period

SW density and properties were compared between *Nlgn2* KO mice and littermates over the course of a 24-h undisturbed recording. A significant interaction between Genotype and Interval was found for SW density (F_34,561_ = 2.9, *p* < 0.001; Fig. 1A), positive phase duration (F_34,561_ = 1.8, *p* < 0.05; Fig. 1E) and frequency (F_34,561_ = 1.7, *p* < 0.05; Fig. 1F). *Nlgn2* KO mice have a higher SW density than WT and HET mice, a difference which was more prominent during the light period. *Nlgn2* KO mice also show a shorter positive phase duration than both WT and HET littermates, which was mainly observed during the dark period, as well as for the first and last 3 intervals of the light period. This observation was paralleled by a higher SW frequency in KO mice in comparison to littermates at similar time intervals. A significant effect of Genotype was found for SW amplitude (F_2,33_ = 10.2, *p* < 0.001; Fig. 1B), slope (F_2,33_ = 12.7, *p* < 0.001; Fig. 1C), and negative phase duration (F_2,33_ = 3.8, *p* < 0.05; Fig. 1D). *Nlgn2* KO mice showed a higher amplitude, steeper slope, and shorter negative phase duration of SW than their littermates, independent of time-of-day. These results indicate a wide range of effects of the KO of *Nlgn2* in male mice on SW density and properties during undisturbed/BL SWS.

### Modified sleep rebound after SD in *Nlgn2* KO mice

**Fig. 2:**
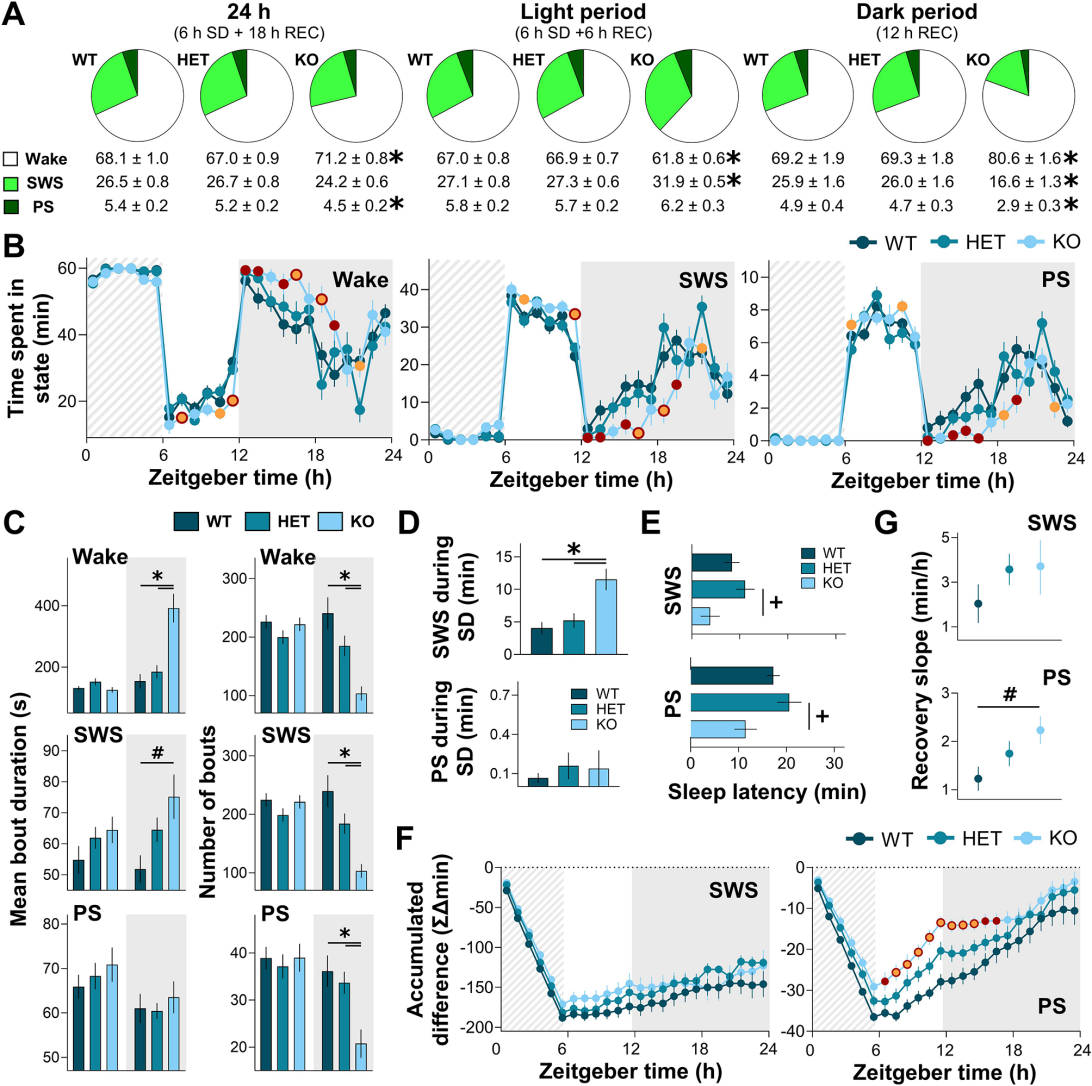
Sleep architecture of *Nlgn2* KO mice and littermates during a 6-h SD and 18-h recovery. **(A)** Percentage of time spent in wake, SWS, and PS for the total 24 h (left), the 12-h light period (middle), and the 12-h dark period (right). **(B)** Twenty-four-hour distribution of time spent in wake, SWS, and PS. **(C)** Mean duration of individual bouts (left) and number of individual bouts (right) of wake, SWS, and PS for the light and dark periods. **(D)** Time spent in SWS (top) and PS (bottom) during the 6-h SD. **(E)** Latency to initiate SWS (top) and PS (bottom) from the end of SD. **(F)** Accumulated difference in SWS (left) and PS (right) during SD/recovery from BL values. **(G)** SWS (top) and PS (bottom) recovery slopes evaluated from the 6th to the 12th intervals of corresponding data in panel F. Significant Genotype by Interval interactions were decomposed by planned comparisons and represented with different color datapoints. Significant Genotype by Light/dark period interactions and main Genotype effects were decomposed by planned comparisons and illustrated with lines accompanied by symbols. *Nlgn2* KO mice red datapoints and # symbols indicate significant differences (*p* < 0.05) in comparison to WT mice. *Nlgn2* KO mice orange datapoints and + signs indicate significant differences (*p* < 0.05) in comparison to HET mice. *Nlgn2* KO mice datapoints in orange with a red contour and stars (*) indicate significant differences (*p* < 0.05) in comparison to both HET and WT mice. Dashed backgrounds represent the 6-h SD. Gray backgrounds represent the dark period. REC: 18-h recovery after SD

The effect of SD on vigilance state duration and distribution was next investigated in *Nlgn2* WT, HET, and KO mice. The percentage of time spent in wakefulness, SWS, and PS was first compared between genotypes for the full 24-h period including the 6-h SD and 18 h of recovery (Fig. 2A, **left**). A significant Genotype effect was found for the percent time spent awake (F_2,36_ = 4.0, *p* < 0.05) and in PS (F_2,36_ = 4.6, *p* < 0.05), while a tendency was found for SWS (F_2,36_ = 3.2, *p* = 0.05). *Nlgn2* KO mice spent a higher percentage of time awake, a lower percentage of time in PS, and tended to spend less time in SWS in comparison to both WT and HET littermates. When specifically considering the light period (Fig. 2A, **middle**), a significant Genotype effect was found for wake (F_2,36_ = 15.6, *p* < 0.001) and SWS (F_2,36_ = 17.4, *p* < 0.001), but not for PS (F_2,36_ = 1.4, *p* = 0.3), showing that *Nlgn2* KO mice spent a lower percentage of time awake and a higher percentage of time in SWS than both WT and HET mice. When considering the subsequent dark period (Fig. 2A, **right**), a significant Genotype effect was found for all states (wake: F_2,36_ = 12.6, *p* < 0.001; SWS: F_2,36_ = 12.4, *p* < 0.001; PS: F_2,36_ = 10.6, *p* < 0.001), showing that KO mice spent a higher percentage of time awake, and a lower percentage of time in both SWS and PS in comparison to WT and HET mice. The 24-h time courses of time spent in each state further support these observations, with significant interactions between Genotype and Hour for wake (F_34,612_ = 2.8, *p* < 0.001; Fig. 2B, **left**), SWS (F_34,612_ = 2.8, *p* < 0.001; Fig. 2B, **middle**), and PS (F_34,612_ = 2.4, *p* < 0.001; Fig. 2B, **right**). *Nlgn2* KO mice spent less time awake and more time asleep across the light period following SD, while the subsequent higher amount of wake (and lower amount of both SWS and PS) observed in these mice is mainly found in the first half of the dark period following SD.

To verify whether effects on time spent in vigilance states were associated with the duration or number of individual bouts of wake, SWS and/or PS, these two variables were investigated separately for the light and dark periods of the SD/recovery 24-h recording. Regarding mean bout duration, a significant interaction between Genotype and Period (light/dark) was found for wake (F_2,36_ = 20.3, *p* < 0.001) and SWS (F_2,36_ = 3.6, *p* < 0.05), but not PS (F_2,36_ = 0.2, *p* = 0.8), showing that *Nlgn2* KO mice had longer wake bouts than WT and HET littermates, and longer SWS bouts than WT mice, but only during the dark period (Fig. 2C, **left**). For the number of bouts, a significant interaction between Genotype and Period (light/dark) was found for wake (F_2,36_ = 13.5, *p* < 0.001), SWS (F_2,36_ = 13.4, *p* < 0.001), and PS (F_2,36_ = 7.0, *p* < 0.05), showing that *Nlgn2* KO mice had less bouts than both littermate groups, again only in the dark period (Fig. 2C, **right**). These results suggest a consolidation of both wake and SWS in KO mice during the dark period. They also indicate that longer wake bout duration and lower number of sleep bouts are the respective main contributors to the increased time spent in wake and decreased time spent in sleep observed in KO mice during the dark period.

The time spent in SWS and PS during the SD was then compared between genotypes to assess potential differences in SD efficacy (Fig. 2D). A significant Genotype effect was found for time spent in SWS during SD (F_2,36_ = 10.4, *p* < 0.001), showing that *Nlgn2* KO mice spent more time in SWS than WT and HET mice. Nevertheless, mice (all genotypes) were deprived of > 94% of their BL sleep amounts during SD, supporting an efficient SD procedure. No Genotype effect was found for PS (F_2,36_ = 0.3, *p* = 0.8). An elevated SWS amount during SD in *Nlgn2* KO mice may result from a shorter sleep latency. The latency to enter SWS or PS after SD was thus compared between genotypes. For SWS, a Genotype effect was found (F_2,36_ = 3.9, *p* = 0.03), showing that *Nlgn2* KO mice initiated SWS faster than HET littermates (trend of *p* = 0.09 for the comparison with WT; Fig. 2E, **top**). For PS, a similar significant Genotype effect was found (F_2,36_ = 4.6, *p* = 0.02), showing that *Nlgn2* KO mice initiated PS faster than HET littermates (trend of *p* = 0.06 in comparison to WT; Fig. 2E, **bottom**). Overall, these results show that *Nlgn2* KO mice tend to fall asleep faster, and this might explain the greater challenge when enforcing wakefulness in these animals.

Sleep loss and recovery were then compared between genotypes using accumulated differences from previously published BL values [24], and this separately for SWS and PS (Fig. 2F). A significant Genotype by Hour interaction was found for the accumulated PS differences (F_34,612_ = 2.3, *p* < 0.05), but not the accumulated SWS differences (F_34,612_ = 1.4, *p* = 0.2). *Nlgn2* KO mice showed smaller accumulated PS differences than WT and HET littermates for approximately 10 h following the end of SD. These findings suggest normal SWS and altered PS recovery dynamics. However, the time spent in a given sleep state during BL affects the accumulated differences during and after SD. To obtain a refined readout of recovery dynamics, we computed the speed of SWS and PS recovery for each genotype. The slope from the 6th to the 12th intervals (recovery specifically occurring during the light period) of accumulated PS difference showed a significant Genotype effect (F_2,36_ = 3.6, *p* < 0.05; Fig. 2G, **bottom**), with *Nlgn2* KO mice recovering PS faster than WT littermates (i.e., steeper slope). No significant Genotype effect was found for the slope of the accumulated SWS difference (F_2,36_ = 1, *p* = 0.4; Fig. 2G, **top**). Altogether, these results indicate that the KO of *Nlgn2* modifies vigilance state duration and distribution following SD in male mice, impacting sleep recovery.

### Altered ECoG response to SD in *Nlgn2* KO mice

**Fig. 3:**
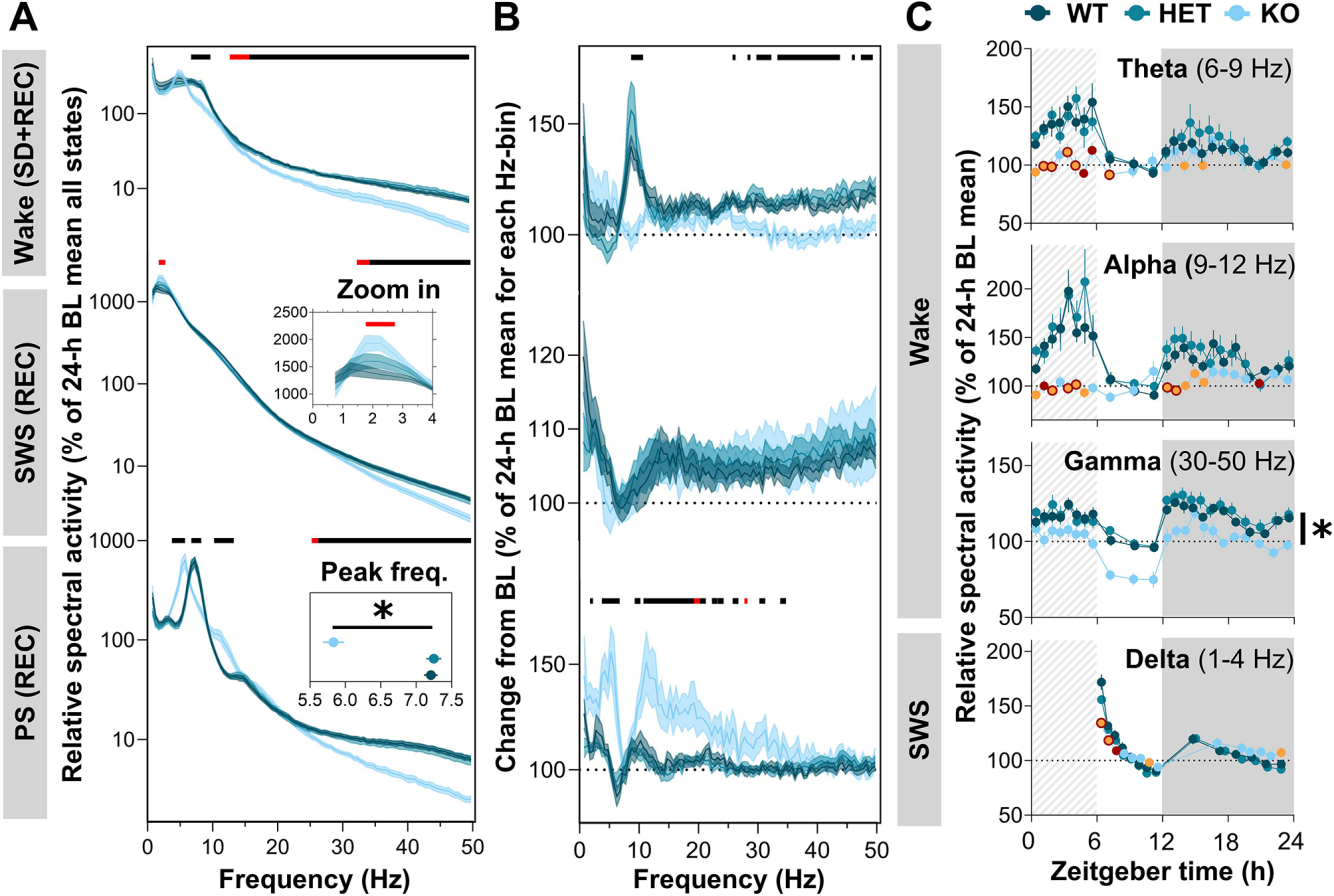
ECoG spectral activity of *Nlgn2* KO mice and littermates during SD and/or recovery. **(A)** Relative spectral activity between 0.5 and 50 Hz during wake (top), SWS (middle), and PS (bottom) for the total 24-h. An enlargement of the SWS activity between 0.5 and 4 Hz is presented in the inset of the middle panel. The peak frequency of PS activity is also presented in the inset of the lower panel. **(B)** Relative spectral activity between 0.5 and 50 Hz during wake (top), SWS (middle), and PS (bottom) for the total 24-h and expressed as percentage of BL **(C)** Twenty-four-hour dynamics of relative activity presented for wake theta, alpha, and gamma frequency bands, and for SWS delta frequencies. Significant Genotype by Interval interactions were decomposed by planned comparisons and represented with different color datapoints. Significant main Genotype effects were decomposed by Tuckey’s post hoc tests in panels A and B, and illustrated with lines at the top of each graphs, or decomposed by planned comparisons in panel C and illustrated with a vertical line accompanied with a star. Red lines at the top of graphs and *Nlgn2* KO red datapoints indicate significant differences (*p* < 0.05) between KO and WT mice. *Nlgn2* KO mice orange datapoints indicate significant differences (*p* < 0.05) in comparison to HET mice. Black lines at the top of graphs, *Nlgn2* KO datapoints in orange with a red contour, and stars (*) indicate significant differences (*p* < 0.05) between KO mice and all littermates. Dashed backgrounds represent the 6-h SD. Gray backgrounds represent the dark period. REC: 18-h recovery after SD

The impact of SD on vigilance state quality (i.e., ECoG spectral content) was explored in *Nlgn2* WT, HET, and KO mice. Wake, SWS, and PS relative spectral activity was compared between genotypes for the 24-h recording including the 6-h SD and 18 h of recovery (Fig. 3A). For wakefulness, significant Genotype effects were found for theta (6.5–10 Hz; F_2,33_ = 5.8–11.1, *p* < 0.05), sigma (12.5–15 Hz; F_2,33_ = 3.4–6.1, *p* < 0.05), beta (15–30 Hz; F_2,33_ = 5.1–20.5, *p* < 0.05), and low gamma (30–50 Hz; F_2,33_ = 10.2–26, *p* < 0.05) frequencies: *Nlgn2* KO mice had less theta, beta, and gamma activity during wake than both WT and HET mice, and less sigma activity than WT mice only (Fig. 3A, **top**). For SWS, significant Genotype effects were found for delta (1.5-3 Hz; F_2,33_ = 3.7–5.8, *p* < 0.05) and low gamma (32–50 Hz; F_2,33_ = 3.3–12.4, *p* < 0.05) frequencies; with *Nlgn2* KO mice having more delta activity than WT, and less low gamma activity in comparison to both WT and HET mice (Fig. 3A, **middle**). Finally, for PS, significant Genotype effects were found for high delta/theta (3.5–8.5 Hz; F_2,33_ = 4.6–32.9, *p* < 0.05), alpha (10-13.5 Hz; F_2,33_ = 6.2–14.3, *p* < 0.05), high beta (25–30 Hz; F_2,33_ = 3.7–14.2, *p* < 0.05), and low gamma (30–50 Hz; F_2,33_ = 13.1–28.9, *p* < 0.05) frequencies: *Nlgn2* KO mice had more low theta and alpha activity, and less high theta, high beta, and low gamma activity than both littermates (Fig. 3A, **bottom**). Interestingly, the PS theta activity alterations in KO mice could result from a shift in the theta-peak frequency, which is at 7.2 $\pm$ 0.1 Hz in WT, 7.3 $\pm$ 0.1 Hz in HET, while at 5.8 $\pm$ 0.1 Hz in KO (F_2,33_ = 45.6, *p* < 0.001; Fig. 3A, bottom-left panel inset).

We previously showed a wide range of ECoG spectral activity changes in *Nlgn2* KO mice under BL conditions [24], which could contribute to the effects reported here during and/or after SD. To examine Genotype differences in SD-induced changes from BL, the power of each Hz-bin was expressed as a percentage of the BL corresponding value for each vigilance state (Fig. 3B). For wakefulness, significant Genotype effects were found for high theta/low alpha (7.5–11 Hz; F_2,33_ = 5.5–13.5, *p* < 0.05) and high beta/low gamma activity (25.5–50 Hz; F_2,33_ = 4.1–18.3, *p* < 0.05; Fig. 3B, **top**). *Nlgn2* KO mice showed a lower SD-induced increase in activity for these frequencies in comparison to littermates. No Genotype effect was found during SWS (F_2,33_ = 0.01–0.6, *p* > 0.05; Fig. 3B, **middle**). For PS, significant Genotype effects were found for delta (1.5–2.5 Hz; F_2,33_ = 3.4–12.1, *p* < 0.05), high delta/low theta (3.5-7 Hz; F_2,33_ = 7.7–17, *p* < 0.05), alpha/sigma (9.5–15 Hz; F_2,33_ = 5.2–13, *p* < 0.05), and beta (15–30 Hz; F_2,33_ = 3.6–11.9, *p* < 0.05) activity, with *Nlgn2* KO mice having a higher SD-induced increase in activity than WT and HET mice (Fig. 3B, **bottom**).

To verify whether the *Nlgn2* mutation impacts the daily dynamics of relative activity in specific ECoG frequency bands during and after SD, the activity of selected frequency bands was computed across a 24-h time course (Fig. 3C). A significant interaction was found between Genotype and Interval for wake theta (6–9 Hz; F_44,594_ = 2.0, *p* < 0.05) and wake alpha (9–12 Hz; F_44,594_ = 2.6, *p* < 0.001), with *Nlgn*2 KO having, in general, less relative activity during the SD and at the beginning of the dark period in comparison to littermates. A significant Genotype effect was rather found for wake gamma (30–50 Hz; F_10,27_ = 10.6, *p* < 0.001) relative activity, with *Nlgn2* KO mice having overall lower activity than both their littermates. A significant interaction was also found between Genotype and Interval for SWS delta (1–4 Hz; F_26,429_ = 7.1, *p* < 0.001) relative activity, with *Nlgn2* KO having mainly less activity than both their littermates for the first two or three intervals following SD. These results indicate that the KO of *Nlgn2* in male mice alters different ECoG responses to SD, with a particularly prominent impact on wake and PS.

### Modified SW properties after SD in *Nlgn2* KO mice

**Fig. 4:**
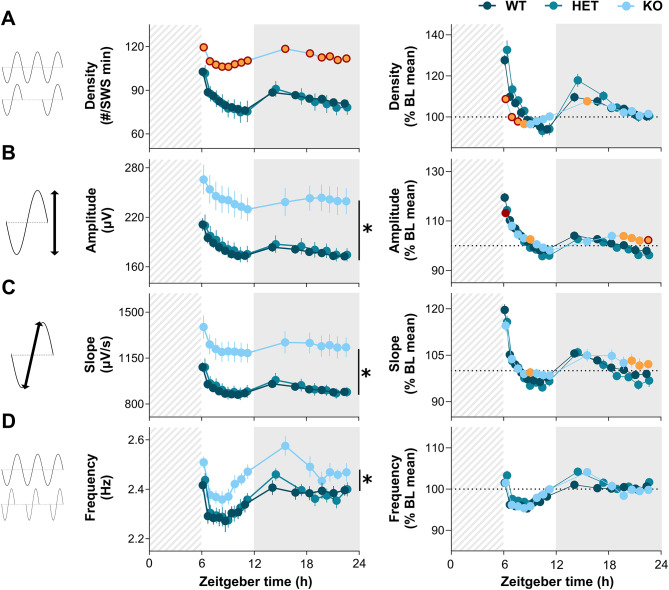
SW density and properties during SWS under recovery conditions in *Nlgn2* KO mice and littermates. Twenty-four-hour dynamics of SW **(A)** density, **(B)** amplitude, **(C)** slope, and **(D)** frequency for the 18-h recovery following a 6-h SD. Left panels show absolute data, and right panels data as a percentage of the 24-h BL mean. A discontinuous line was placed at 100% on the Y axis of right panels to help data visualization. Significant Genotype by Interval interactions were decomposed by planned comparisons and represented with different color datapoints. Significant main Genotype effects were decomposed by planned comparisons and illustrated with vertical lines accompanied by symbols. *Nlgn2* KO red datapoints indicate significant differences (*p* < 0.05) in comparison to WT mice. *Nlgn2* KO orange datapoints indicate significant differences (*p* < 0.05) in comparison to HET mice. *Nlgn2* KO mice datapoints identified in orange with a red contour, as well as stars (*), indicate significant differences in comparison to HET and WT mice (*p* < 0.05). Dashed backgrounds represent the 6-h SD. Gray backgrounds represent the dark period

SW properties such as density, amplitude, and slope have been reported to be higher under elevated homeostatic sleep pressure in both humans and rodents [45, 56–60]. SW density and properties were thus compared between *Nlgn2* KO mice and littermates for the 18 h of recovery following the 6-h SD. A significant interaction was found between Genotype and Interval for SW density (F_26,429_ = 5.8, *p* < 0.001; Fig. 4A, **left**), showing that *Nlgn2* KO mice have a higher SW density than their littermates, with the magnitude of the genotype difference varying across the day. A significant Genotype effect was found for SW amplitude (F_2,33_ = 9.0, *p* < 0.001; Fig. 4B, **left**), slope (F_2,33_ = 10.8, *p* < 0.001; Fig. 4C, **left**), and frequency (F_2,33_ = 4.0, *p* = 0.03; Fig. 4D, **left**). *Nlgn2* KO mice have higher SW amplitude, slope, and frequency than both littermates, independently of time-of-day.

Considering the large between-genotype differences in SW under BL conditions (Fig. 1), SW density and properties were expressed as a percentage of the BL mean values to unmask potential differences in the response to SD. Significant interactions between Genotype and Interval were found for SW density (F_26,429_ = 6.1, *p* < 0.001; Fig. 4A, **right**), amplitude (F_26,429_ = 3.3, *p* < 0.001; Fig. 4B, **right**), and slope (F_26,429_ = 2.0, *p* = 0.01; Fig. 4C, **right**). *Nlgn2* KO mice had a blunted increase in SW density in comparison to littermates for the first three/four intervals following SD. KO mice also had a lower increase in SW amplitude than WT mice for the first interval after SD, and an increase in SW amplitude at the end of the dark period that is absent in both littermates. Finally, *Nlgn2* KO had an increase in SW slope at the end of the dark period which was significantly different in comparison to HET littermates. No significant effect was found for changes relative to BL in SW frequency (Genotype x Interval: F_26,429_ = 1.2, *p* = 0.2; Genotype: F_2,33_ = 1.2, *p* = 0.3; Fig. 4D, **right**). These results indicate that the *Nlgn2* mutation increases SW density, amplitude and slope not only in BL, but also under sleep deprived conditions, and that it generally dampens the 24-h dynamics of SW density and properties.

### SD does not affect hypersynchronized ECoG event occurrence

**Fig. 5:**
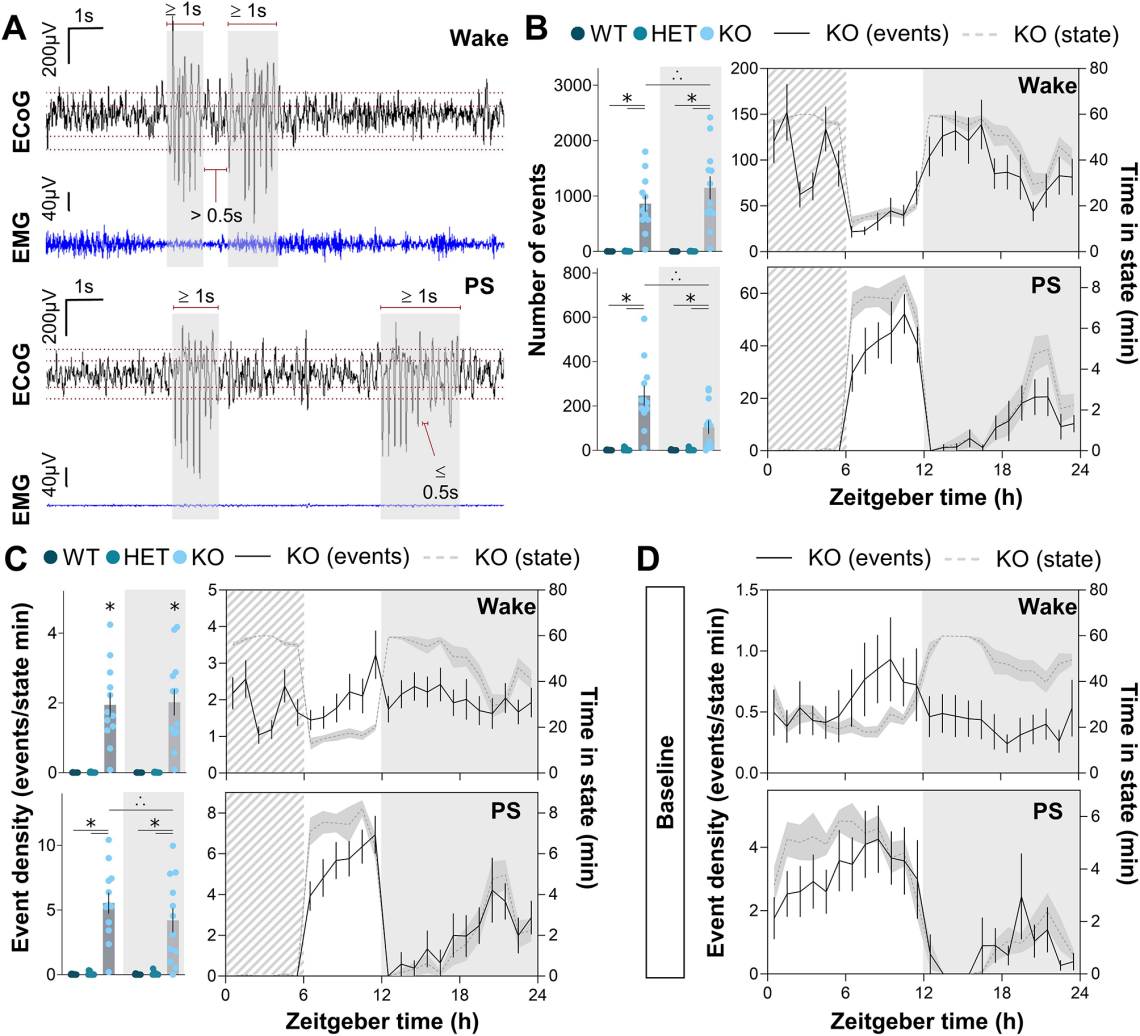
Hypersynchronized ECoG events in *Nlgn2* KO mice and littermates for BL and SD/recovery recordings. **(A)** Examples of wake and PS hypersynchronized events with identification criteria. **(B)** Number of wake (top) and PS (bottom) hypersynchronized events in all genotypes for the light and dark periods (left) of the SD/recovery 24-h recordings. The time courses of event numbers are also shown (right) for KO mice, with time spent in each vigilance state plotted on the right y axes. **(C)** Density of wake (top) and PS (bottom) hypersynchronized events in all genotypes for the light and dark periods (left) of the SD/recovery 24-h recordings. The time courses of event densities are also shown (right) for KO mice, with time spent in each vigilance state plotted on the right y axes. **(D)** Time course of wake (top) and PS (bottom) hypersynchronized events in KO mice for the 24 h of BL with time spent in each state plotted on the right y axes. Significant Genotype by Light/dark period interactions were decomposed by planned comparisons and illustrated with lines accompanied by symbols. Significant Genotype effects are represented by symbols alone. Stars (*) indicate significant differences in comparison to HET and WT mice (*p* < 0.05), and triangles of dots indicate significant light-dark differences (*p* < 0.05). Dashed backgrounds represent the 6-h SD. Gray backgrounds represent the dark period

*Nlgn2* KO mice have hypersynchronized (potentially epileptic-like) ECoG activity [24, 25], and it is known that sleep loss can worsen epileptic manifestations [39]. We thus assessed the effect of SD on hypersynchronized event occurrence. Events were manually identified on the 24-h recordings including 6 h of SD and 18 h of recovery (Fig. 5A). Events were found only during wake and PS, not during SWS, as previously reported [24]. As explained in the Methods, no direct comparisons were made between BL and SD/recovery data to avoid potential scorer bias. It is however possible to assess the effect of homeostatic sleep pressure looking at event progression during and following SD. The number of events was compiled for the light and dark periods for all three genotypes and a significant interaction was found between Genotype and Period (light/dark) for both wake (F_2,36_ = 7.8, *p* = 0.001; Fig. 5B, **top left**) and PS (F_2,36_ = 30.4, *p* < 0.001; Fig. 5B, **bottom left**). Not surprisingly, *Nlgn2* KO mice had more wake and PS events than their littermates, but this difference was higher in the dark period for wake events, and greater in the light period for PS events. Plotting the number of events observed in KO animals in a 24-h time course showed that the number of wake and PS hypersynchronized events follows the same 24-h dynamics as the time spent in each state (Fig. 5B, **right top and bottom**). To better assess an effect of SD on event occurrence, event density was calculated by dividing the number of events in a given state by the time spent in that state. A significant interaction was found between Genotype and Period (light/dark) only for PS (F_2,36_ = 10.7, *p* < 0.001; Fig. 5C, **bottom left**), with *Nlgn2* KO animals having a higher event density than their littermates, but this difference being more prominent in the light than the dark period. Interestingly, only a significant Genotype effect was found for wake hypersynchronized event density (F_2,36_ = 37.1, *p* < 0.001; Fig. 5C, **top left**), with KOs having a greater event density than their WT and HET littermates, independently of the light-dark period. These phenotypes can be further visualized with a 24-h time course, in which wake event density indeed appeared relatively constant throughout the day, while the PS event density seems to peak at the end of both the light and dark periods (Fig. 5C, **top and bottom right**). These results suggest that homeostatic sleep pressure does not increase either wake or PS hypersynchronized event occurrence (e.g., absence of peak at the end of the SD/beginning of recovery sleep).

Still, SD could have modified the 24-h variations of wake and PS hypersynchronized event occurrence compared to BL conditions. To investigate this possibility, data from BL recordings [24] were reanalysed as 24-h time courses of hypersynchronized event densities for wake and PS in *Nlgn2* KO mice (Fig. 5D, **top and bottom**). The 24-h variations in event density during BL appeared relatively similar to those of the SD/recovery recording, with wake event density showing only small changes and PS event density peaking at the light/dark transition. These results suggest no major effect of SD on the overall dynamics of hypersynchronized event occurrence.

### *Nlgn2* is part of the cortical gene network decreased by SD

**Fig. 6:**
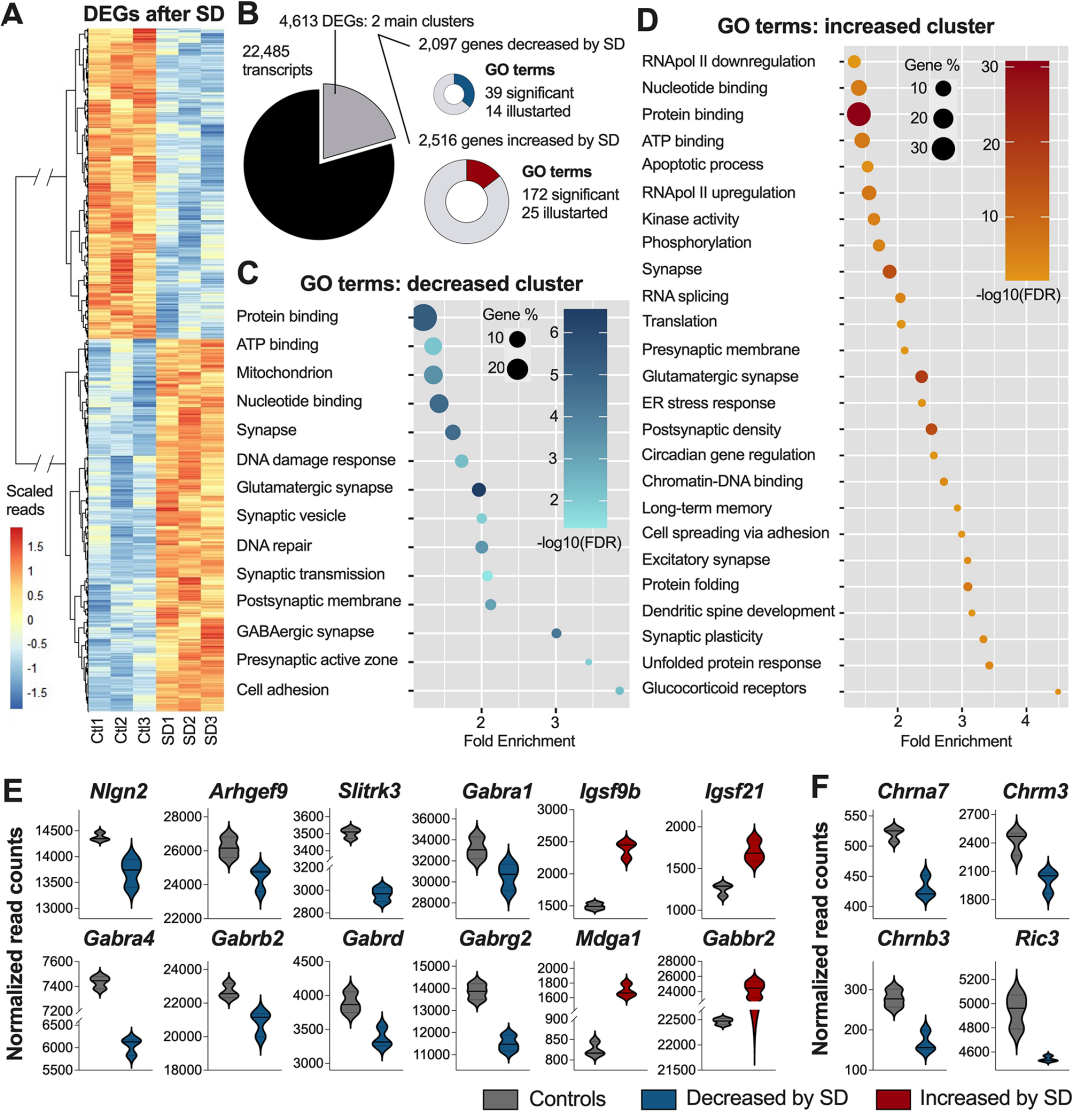
Genome-wide gene expression response to SD quantified for the cerebral cortex of WT mice. **(A)** Hierarchical cluster analysis of DEGs between control and sleep deprived WT mice using Ward’s method. **(B)** Summary of gene expression outputs, number of DEGs, as well as associated GO enrichment. GO term fold enrichment, percentage of genes from the analyzed cluster related to the specific GO terms, and selected GO term FDR for **(C)** decreased and **(D)** increased DEGs. Normalized read counts of DEGs targeted for their association to **(E)** GABAergic and **(F)** cholinergic synapses. DEGs include genes with FDR < 0.05

To explore mechanisms underlying the role of NLGN2 in the homeostatic response to sleep loss, the gene expression response to SD was interrogated in the cerebral cortex of WT mice using RNAseq. Of the 22,485 transcripts for which read number allowed comparison between SD mice and time-matched controls, the expression of 4,613 was significantly changed by SD (FDR < 0.05), which is similar in magnitude to what was reported by previous studies (3,988 transcripts in [61]; 3,201 transcripts in [62]). Hierarchical clustering of DEGs revealed two main clusters: 2,097 transcripts decreased by SD, and 2,516 increased (Fig. 6A). The lists of genes included in each of the two clusters were then separately fed to the DAVID analysis platform for GO analysis, which identifies enriched biological processes, cellular compartments, and molecular functions in each dataset. A total of 39 GO terms were significantly (FDR < 0.05) associated with the cluster of genes with expression decreased by SD, and 172 GO terms were significantly (FDR < 0.05) associated with the cluster of genes with increased expression (Fig. 6B). The transcripts with decreased expression were related to numerous synaptic GO terms including “synaptic transmission”, “presynaptic active zone”, and “synaptic vesicle” (Fig. 6C). Interestingly, “cell adhesion” and “GABAergic synapse”, which are of particular interest regarding NLGN2 functions, were amongst the decreased GO terms with the highest fold enrichment in this dataset (Fig. 6C). Concerning the GO terms enriched in the cluster of transcripts with increased expression, they were related to cellular functions such as transcription/translation (e.g., “RNApol II down/upregulation”, “RNA splicing”, “protein folding”, “chromatin-DNA binding”), stress responses (e.g., “glucocorticoid receptors”, “unfolded protein response”, “ER stress response”, “apoptotic process”), and phosphorylation, as well as to nervous system relevant functions such as “excitatory synapse” and “synaptic plasticity” (Fig. 6D).

Considering the reported involvement of NLGN2 at GABAergic, cholinergic, and dopaminergic synapses [15–18], the effect of SD on the cortical expression of genes involved in these types of neurotransmission was examined in greater detail. As expected from GO enrichment analyses, numerous transcripts related to GABAergic synapses were significantly affected by SD (Fig. 6E). Indeed, SD decreased the cortical RNA levels of *Nlgn2*, *Arhgef9*, *Slitrk3*, *Gabra1*, *Gabra4*, *Gabrb2*, *Gabrd*, and *Gabrg2*, while increasing the levels of *Igsf9b*, *Igsf21*, *Mdga1*, and *Gabbr2* (Fig. 6E). Amongst the decreased transcripts, *Arhgef9* and *Slitrk3* are of particular interest as they encode GABAergic synapse structural proteins that have been shown to interact with NLGN2 [63, 64]. The other decreased GABAergic transcripts (*Gabra1*, *Gabra4*, *Gabrb2*, *Gabrd*, and *Gabrg2*) code for ionotropic GABAergic receptor subunits, while the increased transcript *Gabbr2* encodes a metabotropic GABA receptor. The increased *Mdga1* transcript is also of relevance as it codes for a protein known to prevent NLGN2 binding to its presynaptic ligands the neurexins [65–67]. Finally, the increased *Igsf9b* and *Igsf21* transcripts encode other adhesion proteins involved at GABAergic synapses [68, 69]. In contrast with the large effect of SD on GABAergic transmission-relevant gene expression, only four transcripts related to cholinergic synapse function (*Chrna7*, *Chrnb3*, *Chrm3*, and *Ric3*) were significantly affected by SD, all being decreased (Fig. 6F). As for dopamine neurotransmission-related genes, only the expression of *Comt* was significantly affected by SD, being increased (Control: 2,732 $\pm$ 33 normalized read count; SD: 2,935 $\pm$ 42 normalized read count; FDR = 0.02). Altogether, these results indicate that the expression of a substantial number of genes related to the function of NLGN2 is affected by sleep loss in the mouse cerebral cortex, especially GABAergic neurotransmission-related transcripts.

## Discussion

We here reported striking alterations in the density and properties of individual SW during SWS, as well as in the response to sleep loss in the absence of NLGN2 protein in mice. More specifically, *Nlgn2* KO mice have higher SW density, amplitude, slope, and frequency in comparison to WT and HET mice under BL conditions and following SD. In addition, KO mice spend more time in SWS during early recovery in the light period following SD, more time awake during the subsequent dark period, show an accelerated recovery of lost PS, and an altered ECoG response to sleep loss. Importantly, hypersynchronized wake and PS ECoG events do not peak at the end of the 6-h SD, and the 24-h variations in event occurrence is relatively preserved after SD. In parallel, we have reported that SD decreases the gene expression of *Nlgn2* in the mouse cerebral cortex, together with the expression of several genes associated to GABAergic neurotransmission. Altogether, our findings support a role for NLGN2 in shaping SW during SWS and in modulating the electrophysiological response to sleep loss.

### NLGN2 regulates SW generation and characteristics

Our prior findings of increased SWS delta activity in *Nlgn2* KO mice under BL conditions [24], has led to the hypothesis that KO mice would show increased SW density, amplitude, and/or slope. We here report remarkable changes that support this hypothesis with differences so large that they could be used to differentiate KO animals from WT and HET littermates. A higher SW density can be interpreted as an increase in SW generation. In the current study, SW were detected between frequencies of 0.5 and 4 Hz, which includes both slow oscillations (< 1 Hz) and delta waves (1–4 Hz), or slower (delta 1: 0.75-2 Hz) and faster (delta 2: 2.5–3.75 Hz) delta frequencies as recently studied [70]. Slow oscillations and delta waves appear to originate from different brain networks; slow oscillations being mainly generated by the cerebral cortex and delta waves originating from thalamocortical circuits [70–72]. It is thus likely that the absence of NLGN2 in both intracortical and thalamocortical circuits contributes to the increased SW density observed in *Nlgn2* KO mice. Alterations in inhibitory synaptic transmission in *Nlgn2* KO mice were indeed reported for both the cortex [16, 73, 74] and the ventrobasal thalamus [25]. However, considering our finding of higher SW frequency in the absence of NLGN2 (i.e., mean WT between 2.35 and 2.44 Hz; mean KO between 2.42 and 2.59 Hz), it is expected that the specific generation of delta waves by thalamocortical circuits is particularly boosted in the absence of NLGN2. In parallel, a higher SW amplitude is indicative of a greater number of neurons firing (and being silent) simultaneously, while a steeper SW slope can be interpreted as faster and more synchronous switching between firing and silent states, both signs of higher cortical synchrony [57]. The higher SW amplitude and slope here observed in *Nlgn2* KO mice can thus be interpreted as greater cortical synchronization, as was interpreted before for older mice in comparison to younger ones [75].

One molecular mechanism by which NLGN2 could modulate SW density and properties is through the chloride exporter KCC2. The knockdown of *Nlgn2* was shown to reduce the expression of KCC2 in vitro, which causes a depolarization of GABAergic equilibrium potential [76]. In parallel, chloride transporters (NKCC1 and KCC2) and GABAergic equilibrium potential were recently shown to regulate cortical synchronization and SWS delta activity [77]. For instance, raising intracellular chloride concentration via KCC2 pharmacological inhibition or optogenetic manipulation increases SWS delta activity and neuronal firing during the up state of SW [77]. Accordingly, the KO of *Nlgn2* could boost SWS delta activity and cortical synchronization by reducing KCC2-mediated chloride transport to the extracellular space (and consequently increasing intracellular chloride). This hypothesis could be tested by assessing the effect of a KCC2 activator on SW density/properties in *Nlgn2* KO mice.

### NLGN2 is involved in the electrophysiological response to sleep loss

We also hypothesized that *Nlgn2* KO mice have an altered response to SD, which is supported by our findings showing alterations in sleep rebound and modifications in ECoG activity, including in SD-driven changes in SW density and properties. Regarding sleep architecture, *Nlgn2* KO mice have more SWS than their littermates during the SD, suggesting difficulties to maintain wakefulness under high sleep pressure, and a globally accelerated sleep recovery. We propose that *Nlgn2* KO mice, already spending more time awake under BL conditions [24], present some rigidity/elevated sensitivity to additional sleep pressure such as experienced during SD. These findings could also suggest that different brain regions/neuronal circuits are involved in NLGN2-dependent modulation of normal sleep and of the response to sleep loss, which could be addressed by targeting manipulations of NLGN2 to different sleep-wake regulatory areas such as the basal forebrain, reticular tegmental nuclei or lateral hypothalamus [78].

*Nlgn2* KO mice also showed major alterations in the ECoG response to SD. During SD, WT and HET mice showed a large increase in wake theta-alpha and gamma activity compared to BL, which was almost completely absent in KO mice. One could speculate an implication of the basal forebrain, which projects to the cerebral cortex and contains cholinergic neurons shown to burst maximally during wake and PS, as their bursting correlates with cortical theta and gamma oscillations [78–81]. Moreover, the firing of GABAergic wake-active basal forebrain neurons was also shown to correlate with cortical wake gamma activity [78, 81]. Considering the involvement of NLGN2 at both cholinergic and GABAergic synapses [15, 17], reduced inputs from the basal forebrain might thus be involved in the dampened SD-induced increase in wake theta/gamma activity in *Nlgn2* KO mice. Such altered circuits could also underlie the observed lower PS theta peak frequency, as the firing of PS-active cholinergic basal forebrain neurons specifically correlates with cortical activity around 7 Hz [80].

Interestingly, increased wake theta activity during extended wakefulness was shown to positively correlate with delta activity during recovery sleep in humans [82]. Wake theta and gamma activity were further proposed to be the main drivers for the build-up of homeostatic sleep pressure reflected in SWS delta activity in mice [48]. The absence of theta/gamma-rich wake ECoG activity during SD could therefore greatly contribute to the blunted increases in SWS delta activity and SW density observed in *Nlgn2* KO mice after SD when compared to littermates. A molecular mechanism could again involve KCC2 and intracellular chloride concentration, as the above-mentioned study showed that preventing the shift in GABAergic equilibrium potential after SD reduces the SWS delta activity rebound [77]. It is possible that a downregulation of NLGN2 is required for proper SW rebound, by way of reducing KCC2 function and increasing intracellular chloride. At the gene expression level, we did find a decreased *Nlgn2* expression after SD, which is in line with previous observations [83], and related GABAergic transcripts, which could both contribute to changes in intracellular chloride. Interestingly, a reduced KCC2 expression (mRNA and protein) was reported after SD for the hippocampus in rats [84], and further studies should investigate the mouse cerebral cortex.

### *Nlgn2* KO mouse model relevance to neurodevelopmental disorders

Considering that sleep loss is known to increase epileptic events in patients [39], the third hypothesis of this work was that hypersynchronized ECoG events observed in *Nlgn2* KO mice would increase over the course of SD. This hypothesis was not confirmed, with wake event density being mostly constant through SD and recovery, and PS event density peaking at the light-dark transition. Still, it is possible that sleep loss increases overall 24-h event density in comparison to BL, or changes event duration and spectral composition. However, as the BL and SD/recovery hypersynchronized events were here manually identified, years apart and by different scorers, direct comparison could not be done. An automatic event detector is currently being developed to refine the analysis of this phenotype and ensure reproducible quantification across cohorts/projects.

Nonetheless, the current observations have a notable relevance for neurodevelopmental disorders involving epileptic phenotypes, *NLGN* gene mutations and/or abnormal excitation/inhibition ratio. Epileptic patients were found to have reduced time spent in PS [7, 9, 10], increased SWS delta activity [31, 85], and impaired SW slope dissipation during the sleep episode [30]. These observations are all reminiscent of findings in *Nlgn2* KO male mice, which indicates that understanding the mechanisms by which NLGN2 modulates sleep amounts and quality could help unraveling the cause of comorbid sleep disturbances in the context of neurodevelopmental disorders such as epilepsy. This is also of relevance considering that poor sleep quality has been associated with more frequent/severe seizures and worse quality of life in patients [86, 87]. Future investigations should consider the therapeutic potential of manipulating *NLGN2* expression to improve sleep quality in epileptic patients, including patients with co-morbid ASD.

### Limitations

The main limitation of our work is the inclusion of male mice only, a choice that was made to reduce variability considering the mixed genetic background of the mouse strain studied. Future efforts will however include both sexes as there are known sex differences in both sleep phenotypes [88, 89] and neurodevelopmental disorders prevalence/symptomatology [90–93]. In addition, it is here not possible to differentiate potential neurodevelopmental effects from adult stage-specific phenotypes considering the use of a constitutive *Nlgn2* KO mice. Manipulations of *Nlgn2* expression in young and adult animals will bring further insights into the involvement of NLGN2 in sleep-wake regulation in the context of neurodevelopment.

## Conclusion

We here show that NLGN2 is a key regulator of SW density and properties during SWS, and that its absence affects the response to sleep loss at multiple levels, potentially involving multiple separate sleep regulatory circuits. We specifically uncover that although SW density is strikingly increased in the absence of NLGN2, daily variations and the typical SD-driven increase in SW density are importantly impaired; thus identifying SW density as the main driver of the dampened 24-h dynamics of SWS delta activity previously reported [24]. In parallel, we found that hypersynchronized ECoG events observed in *Nlgn2* KO mice are not changed in the course of SD, and that SD decreases the expression of *Nlgn2* in the cerebral cortex together with a molecular network associated with GABAergic neurotransmission. Given that the current RNAseq data indicate a higher expression of RNA splicing-related transcripts in the cerebral cortex following SD, and that NLGN2 exists in two different isoforms, with one preferentially found at GABAergic synapses [94], investigating the role of different NLGN2 isoforms in sleep regulation will be an important future step.

## Data Availability

The datasets here used and analysed are available from the corresponding author upon reasonable request.

## Abbreviations

ANOVA: analysis of variance
ASD: autism spectrum disorder
BL: baseline
DAVID: Database for Annotation, Visualization and Integrated Discovery
DEG: differentially expressed gene
ECoG: electrocorticographic
EMG: electromyographic
FDR: false discovery rate
GABA: gamma aminobutyric acid
GO: gene ontology
HET: heterozygous
KO: knockout
NLGN: neuroligin
PS: paradoxical sleep
RNAseq: RNA sequencing
SD: sleep deprivation
SW: individual slow wave
SWS: slow-wave sleep
WT: wild-type
ZT: Zeitgeber time (time from light onset)
